# TRPA1 and TRPV1 channels participate in atmospheric-pressure plasma-induced [Ca^2+^]_i_ response

**DOI:** 10.1038/s41598-020-66510-y

**Published:** 2020-06-16

**Authors:** Masayoshi Kawase, Weijian Chen, Kota Kawaguchi, Mazvita R. Nyasha, Shota Sasaki, Hiroyasu Hatakeyama, Toshiro Kaneko, Makoto Kanzaki

**Affiliations:** 1 Graduate School of Biomedical Engineering, Tohoku University, Sendai, Japan; 2 Graduate School of Engineering, Tohoku University, Sendai, Japan; 3 Department of Physiology, Kitasato University School of Medicine, Kitasato, Japan

**Keywords:** Calcium signalling, Molecular medicine

## Abstract

Despite successful clinical application of non-equilibrium atmospheric pressure plasma (APP), the details of the molecular mechanisms underlying APP-inducible biological responses remain ill-defined. We previously reported that exposure of 3T3L1 cells to APP-irradiated buffer raised the cytoplasmic free Ca^2+^ ([Ca^2+^]_i_) concentration by eliciting Ca^2+^ influx in a manner sensitive to transient receptor potential (TRP) channel inhibitors. However, the precise identity of the APP-responsive channel molecule(s) remains unclear. In the present study, we aimed to clarify channel molecule(s) responsible for indirect APP-responsive [Ca^2+^]_i_ rises. siRNA-mediated silencing experiments revealed that TRPA1 and TRPV1 serve as the major APP-responsive Ca^2+^ channels in 3T3L1 cells. Conversely, ectopic expression of either TRPA1 or TRPV1 in APP-unresponsive C2C12 cells actually triggered [Ca^2+^]_i_ elevation in response to indirect APP exposure. Desensitization experiments using 3T3L1 cells revealed APP responsiveness to be markedly suppressed after pretreatment with allyl isothiocyanate or capsaicin, TRPA1 and TRPV1 agonists, respectively. APP exposure also desensitized the cells to these chemical agonists, indicating the existence of a bi-directional heterologous desensitization property of APP-responsive [Ca^2+^]_i_ transients mediated through these TRP channels. Mutational analyses of key cysteine residues in TRPA1 (Cys421, Cys621, Cys641, and Cys665) and in TRPV1 (Cys258, Cys363, and Cys742) have suggested that multiple reactive oxygen and nitrogen species are intricately involved in activation of the channels via a broad range of modifications involving these cysteine residues. Taken together, these observations allow us to conclude that both TRPA1 and TRPV1 channels play a pivotal role in evoking indirect APP-dependent [Ca^2+^]_i_ responses.

## Introduction

Recent innovative plasma technologies allow us to generate non-equilibrium atmospheric pressure plasma (APP), and have thus garnered a great deal of attention due to their biomedical and biotechnical applications. Indeed, direct or indirect application of APP to clinical targets has been successfully employed for wound healing, blood coagulation, the sterilization of surfaces, cancer therapy, and so on^1,2^, though the precise molecular mechanisms underlying these APP-mediated benefits have yet to be elucidated.

While direct application of APP generates charged particles, ultraviolet radiation, electromagnetic fields, and shockwaves^3^, APP also has the capability to produce a variety of reactive oxygen and nitrogen species (RONS), including superoxide radical (O_2_^•−^), peroxynitrite anion (ONOO^−^) and nitric oxide radical (^•^NO) from oxygen (O_2_), nitrogen (N_2_), and water (H_2_O) in ambient air, which can be efficiently delivered into an aqueous biological medium^4^. Given the physiological and pathophysiological importance of RONS in regulating a wide array of biological functions^5,6^, these reactive species generated in the medium via gas-liquid interfacial APPs are regarded as the key factors in the induction of cellular responses to indirect APP treatments; i.e. administration of APP-irradiated medium to the cells^7^. Although there are various cellular proteins that sense RONS and thereby directly contribute to regulating cellular functions^8^, it is increasingly apparent that several members of the transient receptor potential (TRP) channel family, which has been established as a group of sensors of such reactive species^9^, play a crucial role in deciphering the APP-generated reactive species that elicit biological responses by regulating Ca^2+^ influx^10,11^. For example, we recently observed that indirect APP treatment of 3T3L1 fibroblasts results in remarkable and sustained increases in the intracellular free Ca^2+^ concentration ([Ca^2+^]_i_) by eliciting Ca^2+^ influx in a manner that is sensitive to ruthenium red (RR) and SKF96365, both of which are TRP channel inhibitors^11^. Furthermore, these TRP channel inhibitors suppressed APP-inducible enhancement of YOYO-1 uptake into 3T3L1 fibroblasts^12^. Thus, these pharmacological results strongly suggest TRP channel(s) to be intimately involved in the observed APP-inducible biological responses especially in Ca^2+^ influx, though the molecular identity and the activation mechanisms of the APP-responsive channel(s) remain unclear.

In an attempt to clarify channel molecule(s) responsible for the indirect APP-responsive increases in [Ca^2+^]_i_ that were originally detected in 3T3L1 fibroblasts^11^, we carried out two experiments using opposite approaches; 1) siRNA-mediated knockdown experiments of the endogenous APP-responsive Ca^2+^-permeable channels using 3T3L1 fibroblasts, and 2) exogenous expression experiments using APP-unresponsive C2C12 myoblasts. We found that TRPA1 and TRPV1 both function as indirect APP-responsive Ca^2+^-permeable channels.

## Materials and Methods

### Materials

Dulbecco’s modified Eagle’s medium (DMEM), penicillin-streptomycin, and trypsin-EDTA were purchased from Sigma Chemicals (St. Louis, MO). Cell culture equipment was obtained from BD Bioscience (San Jose, CA). Calf serum (CS) and fetal bovine serum (FBS) were obtained from BioWest (Nuaille, France). Expression vectors containing cDNA encoding TRPA1 (FHC07217, pFN21A-TRPA1) and TRPV1 (FHC07221, pFN21A-TRPV1) fused to HaloTag were purchased from Kazusa DNA Research Institute. TRPV1 was subcloned into pFC17K-CMVd3. Mutations were generated by polymerase chain reaction-based site-directed mutagenesis and confirmed by sequencing (PRISM 3130, Applied Biosystems). Scrambled, TRPA1 and TRPV1 siRNAs were purchased from Ambion (Life Technologies Ltd, Japan). The targeting mRNA sequences were as follows;

scrambled, 5′-AGGGUGGGUUUGGCCAAAAtt-3′;

TRPV1, 5′-GGAGUUCACCGAGAACUAUtt-3′;

TRPA1, 5′-CAAUGGAACUAGUAGUACUtt-3′.

siRNAs were introduced into 3T3L1 fibroblasts using Lipofectamine RNAiMax (Thermo Fisher Scientific, MA). Chemical agonists were dissolved in HEPES-buffered saline (HBS) from concentrated stock solutions and delivered via bath application using a syringe-mediated infusion with an overflow aspiration system. A stock solution of capsaicin (33 mM) was made in 10% ethanol, 10% Tween80, and 80% saline. A stock solution of allyl isothiocyanate (AITC; 600 mM) was made in dimethyl sulfoxide (DMSO) and further diluted in HBS to achieve the desired final concentration. Unless otherwise noted, all chemicals were of the purest grade available from Sigma Chemicals or Wako Pure Chemical Industries (Osaka, Japan).

### Plasma irradiation system

A non-equilibrium APP was generated as previously reported^11^. Briefly, helium was used as the working gas, with its flow rate (3 L/min.) through the dielectric tube regulated by a mass flow controller. High-voltage (*V*_*p-p*_) was applied with a frequency of approximately 9 kHz between the two electrodes. The powered electrode was a 1.5-mm diameter tungsten rod and the other was a hot plate, allowing APP to be generated and flow from the nozzle of the quartz glass tube into ambient air. The distance between the bottom edge of the electrode and the edge of the nozzle was 23 mm. The APP was exposed to HEPES-buffered saline (HBS) containing 138 mM NaCl, 5 mM KCl, 0.3 mM KH_2_PO_4_, 4 mM NaHCO_3_, 2 mM CaCl_2_, 1 mM MgCl_2_, and 10 mM HEPES (pH 7.4). The calculated mean power was 1 W for *V*_*p-p*_ = 7.0 kV. Based on our previous reports, HBS was exposed to the APP for 30 sec., we then waited another 30 sec., and finally the APP-irradiated HBS was applied to the Fluo-4-loaded cells via infusion.

### Cell culture

Mouse 3T3L1 fibroblasts (ATCC CL-173) and C2C12 myoblasts^13^ were plated onto 6-well culture plates or glass-bottom dishes (no. 1 S; Matsunami-glass, Osaka, Japan) and cultured in DMEM containing either 10% calf serum (for 3T3L1 fibroblasts) or 10% FBS (for C2C12 myoblasts) at 37 °C under a 5% CO_2_ atmosphere. 3T3L1 fibroblasts were transfected with Lipofectamine RNAiMax (Thermo Fischer Scientific) with 250 pmol siRNAs, and C2C12 myoblasts were transfected with Lipofectamine 3000 (Thermo Fischer Scientific) with 1 μg of plasmid DNAs, according to the manufacturer’s instructions. For siRNA silencing experiments, the transfected 3T3L1 fibroblasts were subjected to experiments at approximately 40 hours after siRNA introduction. For exogenous expression experiments, the transfected C2C12 myoblasts were subjected to experiments at approximately 20 hours after transfection.

### Cytoplasmic calcium ([Ca^2+^]_i_) imaging

3T3L1 fibroblasts were loaded with 5 μM fluo-4 acetoxymethyl ester (Fluo-4AM) (Invitrogen, Carlsbad, CA) and 0.03% Cremophor-EL (C5135, Sigma-Aldrich, St. Louis, MO, USA) in serum-free DMEM for 30 min at 37 °C in an atmosphere of 5% CO_2_^14^. Imaging experiments were performed with an inverted epifluorescence microscope (IX81; Olympus, Tokyo, Japan) equipped with a CCD camera (CoolSNAP; Photometrics), a xenon lamp, and an objective lens (UPlanApo20X, NA0.8, Olympus). Fluo-4 measurements were carried out at room temperature. Fluo-4 fluorescence was acquired through UMNB2 filter cubes (Olympus) every 2 s using illumination periods between 100 and 250 ms in duration for 10–25 min with μManager (http://www.micro-manager.org). The cells were treated with APP-irradiated HBS at 5 and 15 min of acquisition, and then treated with 10 μM ionomycin at ~24 min. Image analysis was performed using Fiji^15^. After subtraction of background fluorescence, changes in [Ca^2+^]_i_ were expressed as *(F* − *F0*)/*F0*, where *F*, and *F0* represent the fluorescence intensity of Fluo-4, and the averaged fluorescence intensity of the dye before stimulation with APP-irradiated HBS, respectively. In some experiments, after acquisition of Fluo-4 fluorescence, the cells expressing HaloTag proteins were stained wih 0.5 μM HaloTag TMR ligand (Promega) for 15 min on site and then a snapshot of TMR fluorescent image was acquired to specify HaloTag-positive cells. We stained the cells with HaloTag TMR ligand afterward to avoid undesirable interference of TMR fluorescence for measuring Fluo-4 fluorescent intensity during [Ca^2+^]_i_ imaging experiments. All experiments were performed at least three times using different batches of cells.

### Subcellular localization of HaloTag-fused TRPA1 and TRPV1 with or without APP-HBS treatment

3T3L1 fibroblasts expressing Halo-TRPA1 or TRPV1-Halo were stained with 0.5 μM HaloTag TMR ligand for 15 min, washed twice with serum-free medium and then incubated for 20 min to remove excess HaloTag TMR ligands. Finally, the cells were treated with or without APP-irradiated HBS for 5 min, fixed with PBS containing 2% paraformaldehyde, and observed with an Olympus FV1000 microscope.

### Quantitative real-time PCR (qRT-PCR) analysis

Total RNA was extracted from 3T3L1 fibroblasts employing TRI reagent (Molecular Research Center Inc., Cincinnati, OH, USA) and was quantified using an ND-1000 spectrophotometer (NanoDrop Tech, Wilmington, DE). cDNA was synthesized using a Transcriptor First Strand cDNA Synthesis Kit with oligo-dT primers (Roche, Basel, Switzerland). Then, qRT-PCR was performed with a Lightcycler 480 SYBR Green reagent and primer mixtures, and detected with a Lightcycler 480 II instrument. The relative expression levels of the target genes were calculated using the 2^–ΔCT^ method with reference genes. Primer sequences were as follows;

Mouse Trpv1 (NM_001001445.2), Forward: 5′-GATGGGCATCTATGCTGTCA-3′, Reverse: 5′-CATCCTCGATCAGTGTCACTAC-3′.

Mouse TRPA1 (NM_177781.5), Forward: 5′-TGGTCCAACATAACCGCATAG-3′, Reverse: 5′-GAATCCATAGGCACACCATTTC-3′. Mouse 36B4 was quantified as a housekeeping gene by using 5′-CGACCTGGAAGTCCAACTAC-3′ and 5′-ATCTGCTGCATCTGCTTG-3′.

### Statistical analysis

The statistical analyses were performed using GraphPad Prism version 7 (GraphPad Software, Inc., La Jolla, CA, USA). All experimental data are presented as means ± S.E. The statistical significance of differences was determined by applying the Dunnett’s multiple comparison test or the Mann-Whitney’s U test. A *p*-value less than 0.05 was taken to indicate a statistically significant difference.

## Results

### [Ca^2+^]_i_ transients induced by indirect APP were significantly blunted by siRNA-mediated knockdown of TRPA1 and/or TRPV1 in 3T3L1 fibroblasts

**Figure 1:**
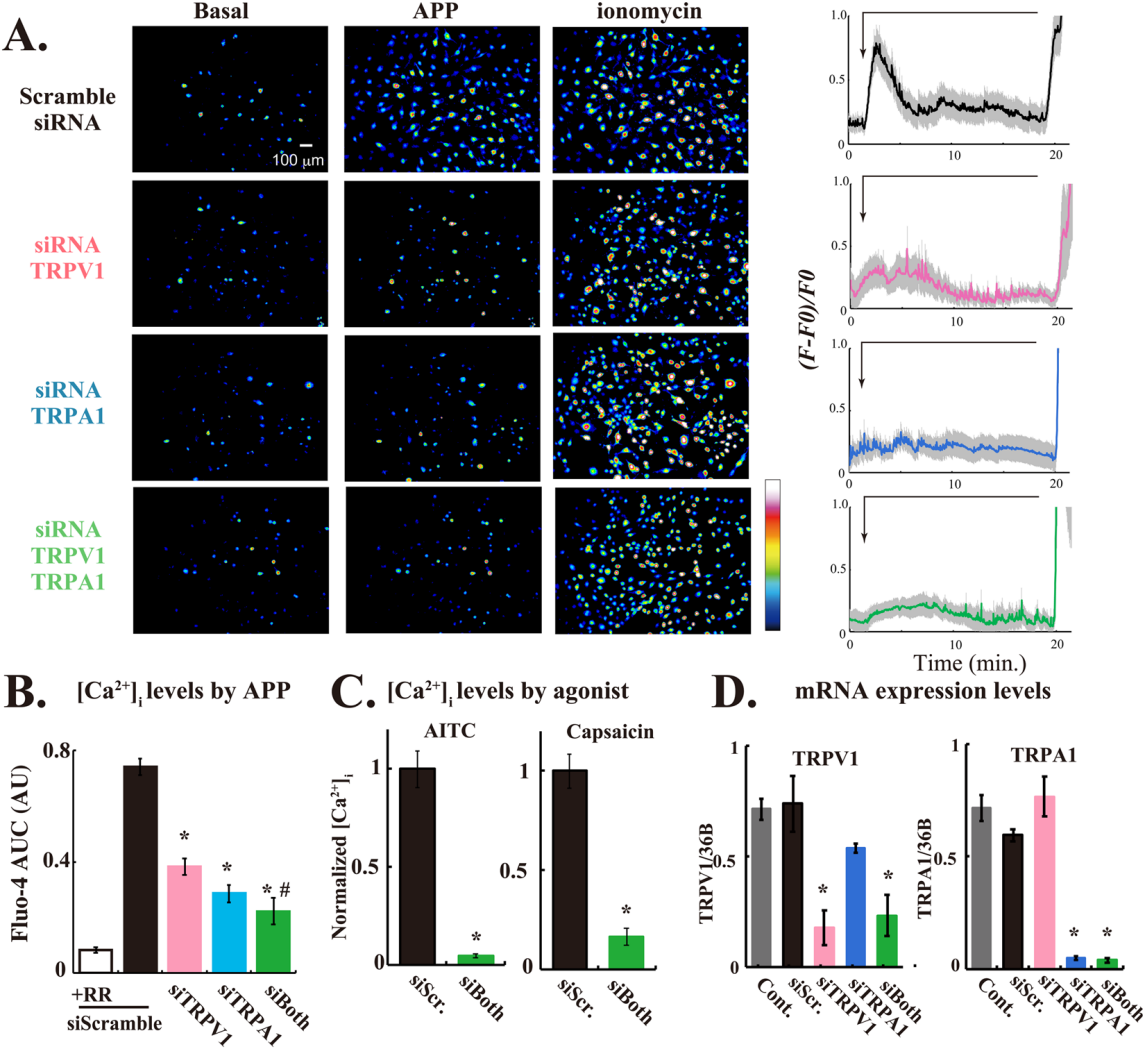
siRNA-mediated TRPV1 and TRPA1 knockdown abrogated the [Ca^2+^]_i_ transients induced by APP-HBS in 3T3L1 fibroblasts. (**A**) Pseudo-color images showing [Ca^2+^]_i_ elevation at ~2 min. after APP-HBS exposure in 3T3L1 cells pretreated with scramble, TRPV1, TRPA1 or TRPV1 plus TRPA1 siRNAs. The cells were stimulated with APP-irradiated HBS at ~1 min. (allows), followed by treatment with ionomycin. The pseudo-color coding on the right shows Fluo-4 fluorescence intensity. The graphs on the right represent mean changes in [Ca^2+^]_i_ within all cells in the images shown on the left, which were expressed as *(F* − *F0)*/*F0* as described in Materials and Methods. The thick colored lines represent the mean values, the shaded region the SE. (**B** and **C**) Quantification of the indirect APP-responsive or agonist-induced [Ca^2+^]_i_ transients obtained from 3–5 independent experiments. For each experiment, more than 50 cells from each glass-bottom dish were measured. (**B**) The area-under-the-curve (AUC) of the *(F − F0*)/*F0* values from 1 to 6 min are shown. (**C**) Normalized [Ca^2+^]_i_ levels of the AUC in siRNA-treated 3T3L1 cells stimulated with AITC or capsaicin are shown. Statistical analysis was performed, versus the control (scramble siRNA), using Dunnett’s multiple comparison and statistical significance is indicated by *(P < 0.05). ^#^Denotes a statistically significant difference (P < 0.05) between the TRPV1 alone and the TRPA1/TRPV1-double knockdown. (**D**) Quantification of TRPV1 and TRPA1 mRNA expression levels in 3T3L1 fibroblasts pretreated with scramble, TRPV1, TRPA1 or TRPV1 plus TRPA1 siRNAs. Statistical significance was determined by applying the Dunnett’s multiple comparison versus the control. The effects of siRNA-mediated knockdown of TRPV1 (pink), or TRPA1 (blue), or both (green) are indicated by *(*P* < 0.05).

Application of APP-irradiated HBS (APP-HBS) induced transient increases in [Ca^2+^]_i_ in 50 to 70% of 3T3L1 fibroblasts (Fig. 1), but these rises were completely abolished in the presence of RR (data not shown), as we previously reported^11^. However, siRNA-mediated knockdown of TRPV1 and/or TRPA1 in 3T3L1 fibroblasts, which was confirmed by RT-PCR analysis (Fig. 1D), markedly inhibited the indirect APP-inducible [Ca^2+^]_i_ transients (Fig. 1A). All values of [Ca^2+^]_i_ transients were calculated using the area under the curve (AUC), and summarized graphically to clearly demonstrate the significant reductions in their indirect APP responsiveness (Fig. 1B). The siRNA-mediated suppression in 3T3L1 fibroblasts of either TRPA1 or TRPV1 expression alone significantly inhibited the indirect APP-dependent [Ca^2+^]_i_ responses. Although the TRPA1/TRPV1 double-knockdown cells exhibited the strongest suppressive response, this suppression did not reach the levels observed in the presence of RR. Pharmacological analyses using AITC (TRPA1 agonist) and capsaicin (TRPV1 agonist) confirmed the existence of functional TRPA1 and TRPV1 channels in 3T3L1 fibroblasts (Fig. 1C), and the agonist-dependent Ca^2+^ influx was significantly inhibited by TRPA1 and TRPV1 double knockdown treatment in 3T3L1 fibroblasts (Fig. 1D). These observations indicate that TRPA1 and TRPV1 serve as the indirect APP-responsive Ca^2+^-permeable channels, both of which are expressed in 3T3L1 fibroblasts.

### Heterologous desensitization of TRPA1 and TRPV1 channels by indirect APP in 3T3L1 fibroblasts

**Figure 2:**
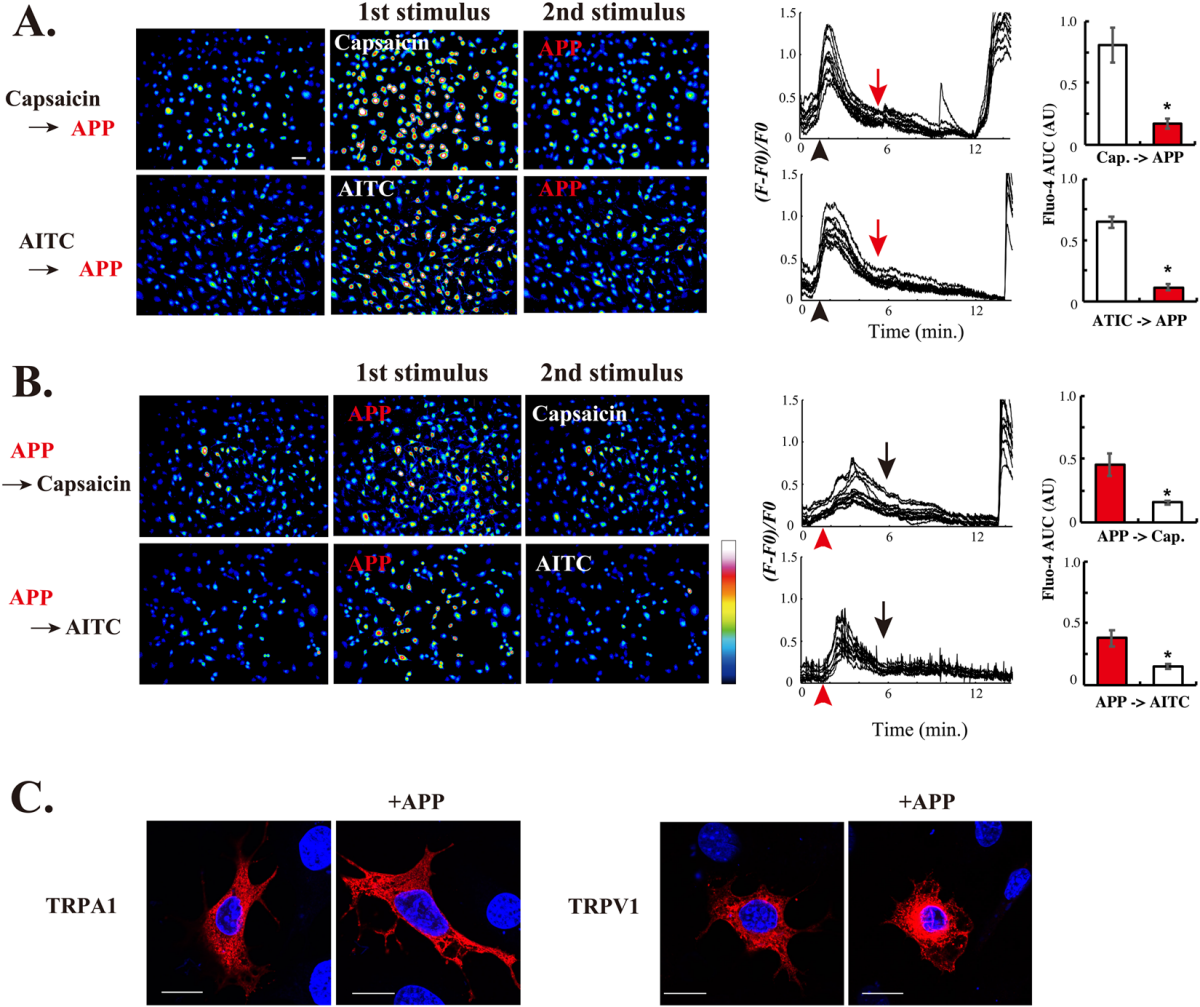
Heterologous desensitization of the indirect APP-responsive [Ca^2+^]_i_ transients in 3T3L1 cells. (**A**) Pseudo-color images showing [Ca^2+^]_i_ elevation in 3T3L1 cells stimulated sequentially with either capsaicin or AITC as the 1^st^ stimulus and then with APP-exposed HBS as the 2^nd^ stimulus. The graphs on the right are representative single-cell traces of Fluo-4 fluorescence from the images on the left, expressed as *(F* − *F0*)/*F0*. Capsaicin or AITC was applied at approximately 1 min. (black arrowheads), and 2.5 mL of APP-HBS were then applied at ~6 min. (red arrows) by infusion. AUC of the *(F* − *F0*)/*F0* values of the 1st stimulus (1–5 min.) and the 2^nd^ stimulus (6–10 min.) are also shown. Scale bar = 100 μm. Statistical significance was determined, versus the 1^st^ stimulus, by the Dunnett’s test. These are representative results obtained from three independent experiments. (**B**) Pseudo-color images showing [Ca^2+^]_i_ elevation in 3T3L1 cells stimulated sequentially with APP-HBS as the 1^st^ stimulus and then with capsaicin or AITC as the 2^nd^ stimulus. The pseudo-color coding on the right shows Fluo-4 fluorescence intensity. Graphs on the right are representative single-cell traces of Fluo-4 fluorescence from the images on the left, expressed as *(F* − *F0*)/*F0*. APP-HBS was applied at approximately 1 min. (*red arrowheads*), and then capcaisin (3 μM) or AITC (100 μM) was administrated at ~6 min. (*black arrows*) by infusion. AUC of the *(F* − *F0*)/*F0* values of the 1st stimulus (1–5 min.) and the 2^nd^ stimulus (6–10 min.) are also shown. Statistical significance was determined, versus the 1^st^ stimulus, by the Dunnett’s test. The results shown are representative of three independent experiments. (**C**) Subcellular localization of Halo-TRPA1 and TRPV1-Halo in 3T3L1 fibroblasts exogenously expressing either Halo-TRPA1 or TRPV1-Halo stimulated with or without APP-HBS for 5 min. The images shown are representative of three independent experiments. Scale bar = 20 μm.

To characterize the indirect APP-responsive regulation of TRP channels, heterologous desensitization experiments were performed by sequentially applying APP-HBS and TRP channel agonists (i.e. AITC for TRPA1 and capsaicin for TRPV1). Treatment with either AITC or capsaicin as the 1^st^ stimulus evoked [Ca^2+^]_i_ transients and the pretreatments markedly inhibited the [Ca^2+^]_i_ responses induced by APP-HBS as the 2^nd^ stimulus (Fig. 2A). Furthermore, pretreatment with APP-HBS resulted in significantly compromised [Ca^2+^]_i_ transients in response to AITC as well as to capsaicin (Fig. 2B). These observations demonstrated the existence of bi-directional heterologous desensitization properties; i.e. chemical agonists, such as AITC or capsaicin, desensitize cells to APP-HBS, and APP-HBS exposure desensitizes the cells to these agonists.

In an attempt to elucidate the mechanism underlying the indirect APP-dependent desensitization, we examined possible changes in subcellular localizations of TRPA1 and TRPV1 channels after APP-HBS exposure. To address this issue, HaloTag-fused TRP channels (i.e. Halo-TRPA1 and TRPV1-Halo) were exogenously expressed and stained with TMR Halo ligands in 3T3L1 fibroblasts. As shown in Fig. 2C, both Halo-TRPA1 and TRPV1-Halo were mostly localized at the intracellular compartments with slight staining of the plasma membrane, and these localization patterns were not detectably changed by APP-HBS treatment, at least with 5–min exposure.

### Exogeneous expression of TRPA1 or TRPV1 generates the indirect APP-responsiveness in C2C12 cells

**Figure 3:**
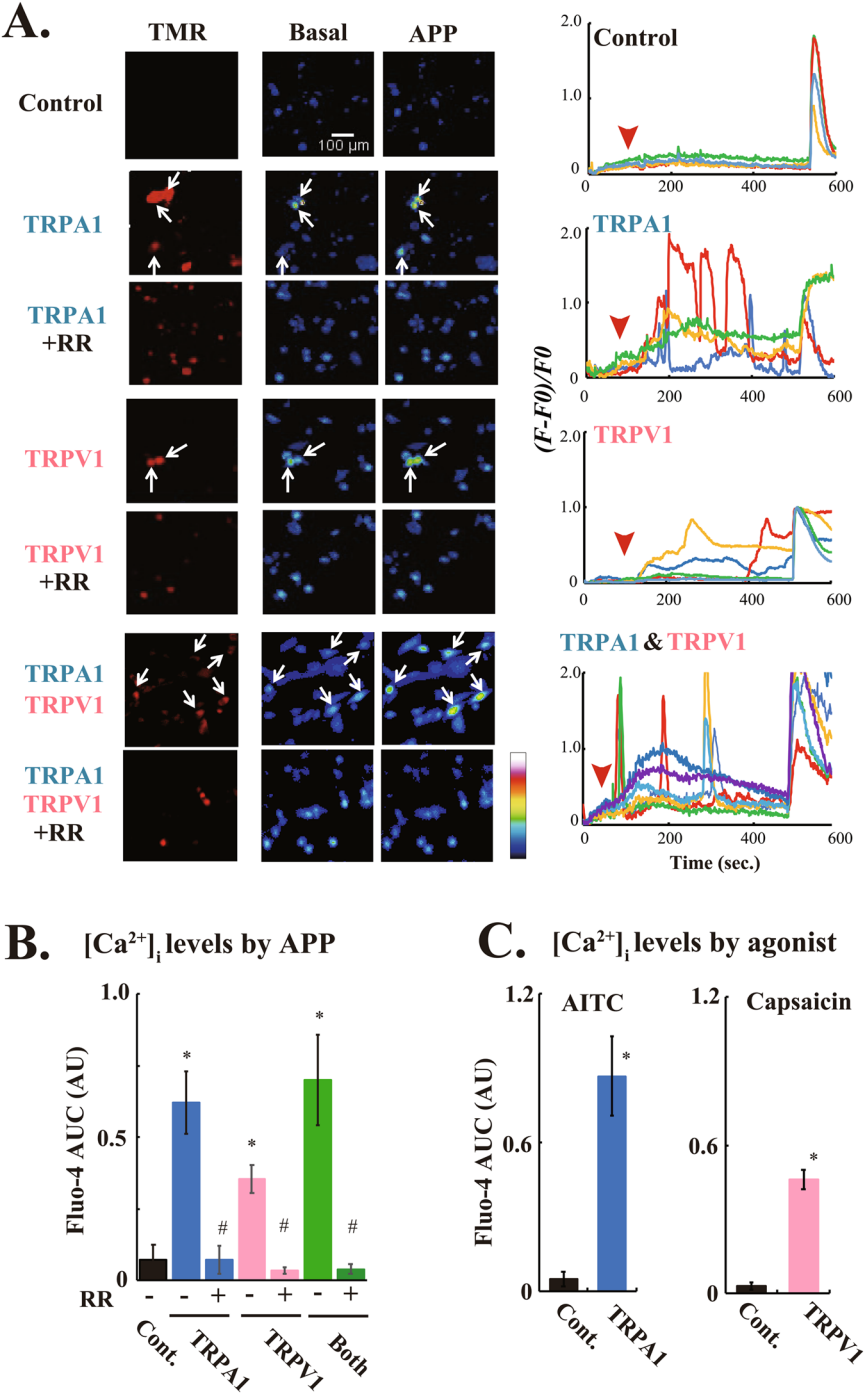
Exogeneous expression of either TRPA1 or TRPV1 generates APP-responsiveness in C2C12 cells. (**A**) Pseudo-color images showing [Ca^2+^]_i_ elevation in C2C12 cells exogenously expressing either Halo-TRPA1 or TRPV1-Halo, alone or in combination (red cells in the left panels) stimulated with APP-HBS in the absence or presence of 100 μM ruthenium red (RR). The graphs on the right are representative single-cell traces of Fluo-4 fluorescence from the experiments shown on the left (-RR only), expressed as *(F − F0*)/*F0*. APP-HBS was applied at approximately 50~100 sec. (*red arrowheads*), and then ionomycin was added. (**B**) Quantification of the indirect APP-responsive [Ca^2+^]_i_ transients in C2C12 myoblasts expressing either TRPA1 (blue bar) or TRPV1 (pink bar), alone or in combination (green bar), with or without 100 μM RR from 3 independent experiments. AUC were evaluated for 300 sec. in total after APP-HBS administration. For each experiment, more than 10 transfected cells (TMR-positive cells) from each glass-bottom dish were measured. Statistical significance was determined by applying the Dunnett’s multiple comparison versus the control (parental C2C12 cells). The effects of exogenous expression of TRPA1 and TRPV1, alone or in combination, are indicated by *(*P* < 0.05), and the effects of RR are indicated by ^#^(*P* < 0.05). (**C**) Quantification of agonist-dependent [Ca^2+^]_i_ transients in C2C12 myoblasts exogenously expressing either TRPA1 (blue bar) or TRPV1 (pink bar) channels from 3 independent experiments. AUC was evaluated from 100–500 sec., and results are expressed as the fold increase versus the AUC obtained from the parental C2C12 cells. For each experiment, more than 10 transfected cells (TMR-positive cells) from each glass-bottom dish were measured. Data are means ± SE., and statistical significance was determined by applying the Mann-Whitney’s U test. The effects of exogenous expression of TRPA1 or TRPV1 channels are indicated by *(*P* < 0.05).

In order to directly address whether TRPA1 and TRPV1 molecules serve as the indirect APP-responsive Ca^2+^-permeable channel, we performed exogenous expression experiments employing Halo-TRPA1 and TRPV1-Halo by using plasma-unresponsive C2C12 myoblasts (Fig. 3). As we previously observed in a human breast cancer cell line, MCF-7 cells^16^, C2C12 myoblasts failed to respond to indirect APP treatment and no rise in [Ca^2+^]_i_ was observed even after APP-HBS administration (Fig. 3A, *upper*). In contrast, exogenous expression of either Halo-TRPA1 or TRPV1-Halo, alone or in combination, in C2C12 myoblasts consistently produced the indirect APP-responsive [Ca^2+^]_i_ transients, which were completely blunted in the presence of RR, a broad spectrum inhibitor for TRP channels^17^ (Fig. 3A,B). Co-expression of TRPA1 and TRPV1 channels did not enhance [Ca^2+^]_i_ transient amplitudes, at least under the present experimental conditions using C2C12 myoblasts. It should be noted that due to the cytotoxicity of TRPV1 possibly due to a robust increase in mitochondrial calcium^18^, a low expression vector (pFC17K-CMVd3) had to be used for exogenous TRPV1 expression experiments using C2C12 myoblasts.

### Mutational analysis of TRPA1 and TRPV1 channels and their indirect APP-responsiveness

**Figure 4:**
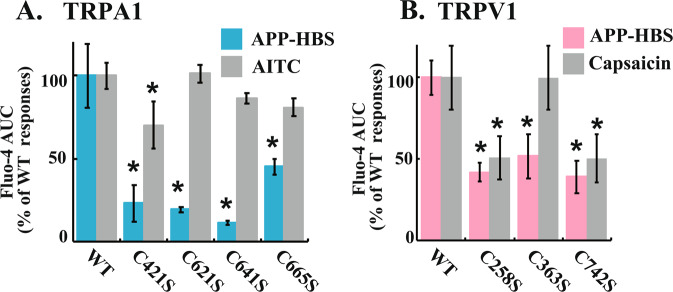
Mutational analysis of TRPA1 and TRPV1 channels for the indirect APP-responsive [Ca^2+^]_i_ transients. (**A**,**B**) Quantification of the indirect APP-responsive [Ca^2+^]_i_ transients as well as the agonist-responsive [Ca^2+^]_i_ transients (*grey bars*) in C2C12 myoblasts expressing either TRPA1/its mutants (*blue bars*) or TRPV1/its mutants (*pink bars*) from 3 independent experiments. APP-HBS was applied at approximately 100 sec., followed by Fluo-4 monitoring for approximately for 10 min as described in Materials and Methods. AUC was evaluated from 100–500 sec. and the results are expressed as a percentage of the data obtained from C2C12 cells expressing wild-type channels. Transfected cells were identified by TMR-conjugated HaloTag-ligand staining after evaluation of [Ca^2+^]_i_ responses by Fluo-4 fluorescence. For each experiment, more than 10 transfected cells (TMR-positive cells) from each glass-bottom dish were measured. Statistical significance was determined by applying the Dunnett’s multiple comparison versus the control (WT). The effects of mutations are indicated by *(*P* < 0.05).

To further understand the indirect APP-responsive regulation of TRPA1 and TRPV1 channels, we generated site-directed mutations of several key cysteine residues in TRPA1^19,20^ and TRPV1^21^ channels and examined their impact on the indirect APP-dependent [Ca^2+^]_i_ responses in C2C12 myoblasts. As shown in Fig. 4, TRPA1/C421S, TRPA1/C621S, TRPA1/C641S and TRPA1/C665S all exhibited markedly compromised [Ca^2+^]_i_ transients in response to APP-HBS as summarized by AUC (Fig. 4A), though TRPA1/C641S showed the most suppressed [Ca^2+^]_i_ responses. These TRPA1 mutants were all responsive to AITC, though TRPA1/C421S exhibited significantly impaired [Ca^2+^]_i_ responses to AITC. Like TRPA1 cysteine mutations, TRPV1/C258S, TRPV1/C363S, and TRPV1/C742S all exhibited reduced [Ca^2+^]_i_ transients in response to the indirect APP **(**Fig. 4B), and no further reduction was observed with its double and triple mutants (data not shown), though TRPV1/C258S and TRPV1/C742S, but not TRPV1/C363S, exhibited impaired [Ca^2+^]_i_ transients in response to capsaicin.

## Discussion

A central issue in understanding biological impacts of APP is elucidation of the molecular mechanisms allowing cells to decipher and respond to the physiologically-relevant, but not excessive and non-beneficial, levels of APP exposure. By employing cell-based [Ca^2+^]_i_ analyses of both siRNA-mediated silencing (Figs. 1 and 2) and exogenous expression strategies (Figs. 3 and 4), we demonstrated both TRPA1 and TRPV1 channels to be molecules responsible for indirect APP-dependent [Ca^2+^]_i_ response. Our data also revealed that the indirect APP-responsive [Ca^2+^]_i_ transients exhibited a bi-directional heterologous desensitization property with either TRPA1 or TRPV1 activation by their agonists (Fig. 2**)**. Furthermore, mutational analysis of key cysteine residues in TRPA1 (Cys421, Cys621, Cys641, and Cys665) and TRPV1 (Cys258, Cys363, and Cys742) exogenously expressed in C2C12 myoblasts strongly suggests that the cysteine residues of TRPA1 and TRPV1 channels play a crucial role in the indirect APP-responsive regulation of channel activation (Fig. 4). While it is possible that other types of channels responsive to APP may exist^22^, our findings provide pivotal insights into the molecular mechanisms that underlie the indirect APP-dependent [Ca^2+^]_i_ transients, thereby further extending the potential of plasma medicine based on APP technology.

Consistently with several reports showing that 3T3L1 fibroblasts endogenously express multiple TRP channel family members including TRPA1 and TRPV1^23,24^, we observed that 3T3L1 fibroblasts exhibit rises in [Ca^2+^]_i_ in response not only to APP-HBS exposure but also to either AITC or capsaicin, pharmacological activators of TRPA1 and TRPV1, respectively. Consequently, we confirmed all of these [Ca^2+^]_i_ responses induced by APP-HBS as well as by these chemical agonists to be significantly suppressed by siRNA-mediated knockdown of TRPA1 and/or TRPV1 (Fig. 1). It has now been well established that both TRPA1 and TRPV1 channels, in addition to their temperature-sensing property, serve as redox-sensitive TRP channels that can be activated by environmental and endogenous RONS^9,25^. In particular, TRPA1 and TRPV1 are reportedly activated by hydrogen peroxide (H_2_O_2_), OH^•^, NO and ONOO^−^ with varying sensitivities^9,26–29^. Considering the highly efficient capability of APP for producing OH^•^, O_2_^•−^, H_2_O_2_, ONOO^−^, ^•^NO, and so on, in an APP-irradiated solution^4,30^, it is evident that multiple RONS generated in the APP-HBS, not only alone but also perhaps collectively, participate in exerting the entire range of APP-dependent [Ca^2+^]_i_ responses by modulating channel activities of both TRPA1 and TRPV1 in 3T3L1 fibroblasts.

We have not as yet defined the precise reactive species directly involved in the activation of TRPA1 and TRPV1 channels, but the findings of our previous study^11^ strongly suggest the involvement of relatively short-lived reactive species (i.e. deactivated within approximately 10 min) generated in the APP-HBS in response to APP irradiation. In this regard, a recent report demonstrated that alginate solution and hydrogels show a capacity for sustained release of reactive species^31^. Furthermore, we have recently found that HEPES, an amine-based buffer compound with a piperazine skeleton, to play an important role in the cycling chain reaction of chemically reactive species, which is initiated by the APP-generated hydroxyl radicals, resulting in continuous release of O_2_^•−^/ONOO^−^ in the APP-irradiated HBS which persists for several minutes, at least^32^. Thus, despite our general former understanding that O_2_^•−^, ^•^NO, and ONOO^−^ have very short half-lives (less than one second) in biological solutions, these highly potent reactive species, both biologically and chemically, may be directly involved in the activation processes of both TRPA1 and TRPV1 channels when certain compounds exist in the solution. Future work is necessary to clarify the precise molecular interactions between these APP-generated reactive species and TRPA1/TRPV1 channels under conditions wherein the aforementioned reactive species are experimentally manipulated in various ways allowing the roles of individual molecules to be demonstrated.

Given that exogenous expression of either TRPA1 or TRPV1, alone or in combination, actually endowed C2C12 cells with APP-responsiveness (Fig. 3), it is obvious that each molecule individually possesses a property that can be independently activated by indirect APP exposure. Intriguingly, however, siRNA-mediated knockdown of either TRPA1 alone or TRPV1 alone in 3T3L1 fibroblasts significantly suppressed indirect APP-responsive [Ca^2+^]_i_ transients, even though the highest degrees of suppression was observed when both TRPA1 and TRPV1 were depleted (Fig. 1). These results suggest that the [Ca^2+^]_i_ rises in response to APP-HBS could also be mediated through functional interactions between endogenous TRPA1 and TRPV1 channels in 3T3L1 fibroblasts. In this regard, several lines of evidence point to TRPA1 and TRPV1 being able to form physical and functional heteromeric channel complexes possessing synergistic activation properties^33–35^. For example, a combined application of AITC and capsaicin reportedly resulted in significantly longer durations of responses in isolated rat vagal pulmonary sensory neurons, which were dependent upon extracellular Ca^2+,^^36^. Moreover, potentiating effects were also reportedly generated when capsaicin was replaced by high temperature (>39 °C), a natural biological activator of TRPV1^36^. Furthermore, TRPA1/TRPV1-interacting proteins, such as Tmem100, AKAP and Toll-like receptor 4, have been reported to modulate TRPA1 and/or TRPV1 channels^37–39^. Thus, while the TRPA1 or TRPV1 channels can each be independently activated by administration of APP-HBS, it is likely that indirect APP-responsive [Ca^2+^]_i_ transients in 3T3L1 fibroblasts were induced by cooperative actions of TRPA1 and TRPV1 channels possibly along with the other aforementioned interacting protein(s) and their splicing variant forms^40,41^. These intricate regulatory mechanisms including the participation of such interacting proteins, depending on cellular contexts, might contribute to the observed differences in the profiles of the APP-responsive [Ca^2+^]_i_ transients between ectopically expressed C2C12 cells and naive 3T3L1 fibroblasts.

Consistently with our findings depicted in Fig. 2, heterologous desensitization of TRPA1 and TRPV1 by their pharmacological agonists including AITC and capsaicin has been reported in endogenous neurons and in heterologous expression systems^42–45^. In this regard, it is well-established that highly increased [Ca^2+^]_i_ levels in response to pretreatments with pharmacological agonists resulted in a Ca^2+^-dependent desensitization of the channel itself at the molecular level via multiple mechanisms involving protein kinase A, protein kinase C, Ca^2+^/Calmodulin-dependent kinase II and/or calcineurin^35,42,44^. Thus, relatively high increases in [Ca^2+^]_i_ levels upon application of the initial stimulus with either AITC or capsaicin may contribute to the desensitization phenomenon observed with subsequent APP-HBS exposure (Fig. 2A). In addition, several lines of evidence indicate that endocytosis of TRPV1 channels might be involved in the capsaicin-dependent desensitization process^46–48^. As shown in Fig. 2C, given that no obvious alterations in subcellular localizations of both TRPA1 and TRPV1 upon APP-HBS exposure were observed, this appears to not be the case for APP-induced desensitization. It is also possible that the extrusion activity of [Ca^2+^]_i_ by Ca^2+^ ATPase^49,50^ could be impacted by this indirect APP treatment.

Thus, although the molecular mechanisms underlying the indirect APP-dependent cooperative activation of as well as the desensitization of TRPA1 and TRPV1 are not understood in detail, and the functional properties of the two channels in combination in response to APP-HBS treatment require further elucidation, our present data provide important insights into the mode of APP-responsive [Ca^2+^]_i_ rises through TRPA1 and TRPV1 channels in 3T3L1 fibroblasts.

Finally, mutational analyses of several key cysteine residues in TRPA1 and TRPV1 provide compelling evidence that cysteine residues play crucial roles in their channel activation in response to the APP-irradiated HBS containing various reactive species that have yet to be clarified (Fig. 4). Given that all cysteine mutants of either TRPA1 (C421S, C621S, C641S, C665S) or TRPV1 (C258S, C363S, C742S) clearly suppressed the indirect APP-inducible [Ca^2+^]_i_ transients, though there were slight variations among these mutants (e.g. TRPA1/C665S), the precise molecular details of their channel activation processes upon exposure to APP-irradiated HBS remain uncertain at present. Nevertheless, some mutant forms of TRPA1 (i.e. TRPA1/C421S) and TRPV1 (i.e. TRPV1/C258S and TRPV1/C742S) also exhibited impaired agonist-dependent [Ca^2+^]_i_ transients.

It has been well established that modifications of several key cysteine residues of both TRPA1 and TRPV1 channels, including their oxidation, electrophilic interactions, and S-nitrosylation of sulfhydryls, are among the most common mechanisms for channel activations in response to a wide array of stimuli^51^. As an example, TRPA1, Cys633, Cys641, and Cys665 are reportedly crucial for activation by electrophiles^52,53^, and Cys641 and Cys665 have been shown to be involved in channel activation processes evoked by H_2_O_2_ and NO^54,55^. Moreover, disulfide formation between Cys621 and the neighboring Cys633 as well as that between Cys651 and Cys665 have been shown to play important roles in the activation elicited by HNO, the one-electron reduced form of NO^56^. Several lines of evidence have also demonstrated a crucial role of Cys641 in the Pro394 hydroxylation-dependent channel modulation via inhibition of prolyl hydroxylase, which apparently sensitizes TRPA1 to reactive oxygen species^19^. For instance in TRPV1, Cys258 and C363 are reportedly involved in H_2_O_2_-dependent activation^21^. Furthermore, it has been reported that Cys258 and Cys742, which are located near each other, are heterogeneously modified to form disulfide bonds in the TRPV1 tetrameric complexes facilitating subunit dimerization^21^. Thus, while each cysteine residue appears to individually contribute to stimulus-specific responses to some extent, functions of these cysteine residues are related to each other in complex ways, even within either the TRPA1 or the TRPV1 molecule alone, obviously adding a further layer of complexity when both are expressed in a cell. Based on these considerations, our mutation analysis, displaying all examined cysteine mutants with phenotypes associated with suppressive actions in response to indirect APP, strongly suggested that multiple reactive species which vary over time are intricately involved in the activation processes of both TRPA1 and TRPV1 channels. This complex interplay presumably occurs via a broad range of modifications of these cysteine residues. Furthermore, our findings using these various mutants raised the clear possibility that APP-dependent desensitization (Fig. 2B) might be attributable to covalent modifications of these cysteine residues, which could be promiscuous (non-physiological), by multiple RNOS including OH^•^, O_2_^•−^, H_2_O_2_, ONOO^−^, ^•^NO, and their derivatives in the APP-irradiated solution. Indeed, desensitization of TRPA1 channels and their covalent modifications involving several cysteine residues by mastered oil, another reactive electrophilic chemical compound, reportedly occurred even in the absence of Ca^2+^ ^44,57^. Future studies employing more sophisticated approaches are clearly required to elucidate the mechanistic details of the APP-responsive activation as well as the desensitization of these channels.

In summary, we herein provide strong evidence that both TRPA1 and TRPV1 channels play a pivotal role in evoking indirect APP-dependent [Ca^2+^]_i_ responses. Since both TRPA1 and TRPV1 have received considerable attention as potential therapeutic targets for the treatment of several disorders including chronic pain, inflammation, respiratory diseases, and cancers^58–60^, further detailed mechanistic analyses of the APP-responsive activations of TRPA1 and TRPV1 channels is anticipated to provide new research insights for developing therapeutic interventions applicable to plasma medicine.
